# Supplementary material for: Analysis of arsenic-modulated expression of hypothalamic estrogen receptor, thyroid receptor, and peroxisome proliferator-activated receptor gamma mRNA and simultaneous mitochondrial morphology and respiration rates in the mouse
Source: PLoS One. 2024 May 16;19(5):e0303528. doi: 10.1371/journal.pone.0303528 (PMC11098319; doi:10.1371/journal.pone.0303528)
Supplement: Attachment — Submitted filename: PLOS_RESPONSE_RVW2_FINAL.docx [file pone.0303528.s004.docx]

Response to Reviewer 2 Comments

**PONE-D-23-41249
*"Analysis of arsenic-modulated expression of hypothalamic estrogen receptor, thyroid receptor, and peroxisome proliferator-activated receptor gamma mRNA and simultaneous mitochondrial morphology and respiration rates in the mouse."***

**PLOS ONE**

We appreciate the time and effort of Reviewer 2 to dedicate his/her time in evaluating our manuscript. We have addressed all issues indicated in the review report, and hope that the revised version meets the journal publication requirements.

**Question 1:** ***“40 µg, 5 mg, and 10 mg- please explain the choose and calculation.”***

**Answer:** Thank you for your response. We have taken your feedback into account and made expanded modifications to the text accordingly, (please, see lines 118-133).

**Question 2:** ***“Provide histological microscopic documentation.”***

**Answer:** Thank you for your query. We have carefully reviewed the manuscript, and the histological documentation in the form of electron microscopic images (please, see Figure 3 G, H) has already been provided in the study. However, to ensure clarity and address your request, could you please elaborate on the specific type of histological documentation you are referring to? Any additional details you can provide will help us address your concerns effectively. We can insert more photos upon request.

**Question 3:** ***“50 µm thick tissue slices for ihc ?give robust explanation.”***

**Answer:** We sincerely appreciate the reviewer's attention to detail and valuable feedback. The choice of 50 µm thick tissue slices for IHC is based on careful considerations of tissue morphology preservation and staining efficiency. Tissue sections within the range of 20-50 μm thickness (Tremblay et al., 2010; DOI: doi:10.3791/2021; Potts et al., 2021; doi: 10.3791/61622; Partida et al., 2023; https://doi.org/10.3389/fnana.2023.1154568); are generally selected to ensure the retention of structural integrity and cellular morphology essential for accurate histological analysis. Specifically, when employing 4% paraformaldehyde fixation, the optimal thickness falls within the range of 20-50 µm for floating sections, facilitating efficient penetration of antibodies and subsequent antigen retrieval. Subsequently, processing to electron microscopy, ultrathin sections at a thickness of 50 nm were cut, ensuring precision, (please see the modified text at lines 208-211; 309-312).

**Question 4:** ***“******The age chosen represents a pre-pubertal state when the hypothalamus …” add citation.”***

**Answer:** Thank you for your suggestion. We have noted your request and have added the appropriate citation to support this statement (please, see lines 165-171).

**Question 5:** **“*Add study limitations.”***

**Answer:** Study limitations can be formulated from several perspectives. Firstly, our study primarily focused on acute (immediate) effects of arsenic exposure following a single intraperitoneal injection. Understanding the sustained impact of arsenic over extended periods could provide valuable insights into its cumulative effects on neuroendocrine regulation.

Secondly, although we observed dose-dependent alterations in mitochondrial respiration and morphology, our study did not delve into the underlying mechanisms driving these changes. Further investigations elucidating the molecular pathways involved would enhance our understanding of arsenic-induced mitochondrial dysfunction.

Additionally, our study primarily examined hypothalamic tissue homogenates, providing a global view of gene expression and mitochondrial function.
